# The longitudinal relation between executive functioning and multilayer network topology in glioma patients

**DOI:** 10.1007/s11682-023-00770-w

**Published:** 2023-04-17

**Authors:** Marike R. van Lingen, Lucas C. Breedt, Jeroen J.G. Geurts, Arjan Hillebrand, Martin Klein, Mathilde C.M. Kouwenhoven, Shanna D. Kulik, Jaap C. Reijneveld, Cornelis J. Stam, Philip C. De Witt Hamer, Mona L.M. Zimmermann, Fernando A.N. Santos, Linda Douw

**Affiliations:** 1 Department of Anatomy and Neurosciences, Amsterdam UMC location Vrije Universiteit Amsterdam, de Boelelaan 1108, Amsterdam, the Netherlands; 2 Amsterdam Neuroscience, Brain Imaging, Amsterdam, the Netherlands; 3 Amsterdam Neuroscience, Systems & Network Neurosciences, Amsterdam, the Netherlands; 4 Department of Clinical Neurophysiology and MEG Center, Amsterdam UMC location Vrije Universiteit Amsterdam, De Boelelaan 1117, Amsterdam, the Netherlands; 5 Department of Medical Psychology, Amsterdam UMC location Vrije Universiteit Amsterdam, De Boelelaan 1117, Amsterdam, the Netherlands; 6 Department of Neurology, Amsterdam UMC location Vrije Universiteit Amsterdam, De Boelelaan 1117, Amsterdam, the Netherlands; 7 Stichting Epilepsie Instellingen Nederland (SEIN), Heemstede, the Netherlands; 8 Department of Neurosurgery, Amsterdam UMC location Vrije Universiteit Amsterdam, De Boelelaan 1117, Amsterdam, the Netherlands; 9 Institute of Advanced Studies, University of Amsterdam, Amsterdam, the Netherlands; 10 Cancer Center Amsterdam, Amsterdam, the Netherlands

**Keywords:** Graph theory, Network neuroscience, Functional connectivity, Eigenvector centrality, Cognition

## Abstract

Many patients with glioma, primary brain tumors, suffer from poorly understood executive functioning deficits before and/or after tumor resection. We aimed to test whether frontoparietal network centrality of multilayer networks, allowing for integration across multiple frequencies, relates to and predicts executive functioning in glioma. Patients with glioma (n = 37) underwent resting-state magnetoencephalography and neuropsychological tests assessing word fluency, inhibition, and set shifting before (T1) and one year after tumor resection (T2). We constructed binary multilayer networks comprising six layers, with each layer representing frequency-specific functional connectivity between source-localized time series of 78 cortical regions. Average frontoparietal network multilayer eigenvector centrality, a measure for network integration, was calculated at both time points. Regression analyses were used to investigate associations with executive functioning. At T1, lower multilayer integration (*p =* 0.017) and epilepsy (*p =* 0.006) associated with poorer set shifting (adj. *R*^*2*^ = 0.269). Decreasing multilayer integration (*p =* 0.022) and not undergoing chemotherapy at T2 (*p =* 0.004) related to deteriorating set shifting over time (adj. *R*^*2*^ = 0.283). No significant associations were found for word fluency or inhibition, nor did T1 multilayer integration predict changes in executive functioning. As expected, our results establish multilayer integration of the frontoparietal network as a cross-sectional and longitudinal correlate of executive functioning in glioma patients. However, multilayer integration did not predict postoperative changes in executive functioning, which together with the fact that this correlate is also found in health and other diseases, limits its specific clinical relevance in glioma.

**Supplementary Information:**

The online version contains supplementary material available at 10.1007/s11682-023-00770-w.

## Introduction

Gliomas, originating from glial cells, are the most common primary brain tumors and are fatal. Despite their local appearance on MRI, patients experience varied cognitive complaints that cannot be attributed to location alone (De Baene et al., 2019; van Kessel et al., 2017). Executive functioning (EF), including higher-order cognitive processes such as planning, working memory, inhibition and flexibility, is often affected at diagnosis, before treatment (Noll et al., 2020; Tanzilli et al., 2022; van Kessel et al., 2017), and may contribute to lower quality of life (Weyer-Jamora et al., 2021). Subsequently, there is large individual variability in cognitive trajectories across the disease course. After tumor resection, improving, stable, and deteriorating EF is observed (Lemaitre et al., 2021; Ng et al., 2019; Noll et al., 2015; Satoer et al., 2012; Sinha et al., 2020; Tabor et al., 2021; Talacchi et al., 2011; Wu et al., 2011). Many patients also receive chemo- and/or radiotherapy, depending on tumor subtype and residual tumor, which impacts EF both positively and negatively according to the literature (Hilverda et al., 2010; Koutsarnakis et al., 2021; Tanzilli et al., 2022). Other relevant correlates of poorer EF are higher age, lower Karnofsky performance status (KPS), frontal tumor location (Fang et al., 2014), and use of antiepileptic drugs (Klein et al., 2004). However, it remains impossible to predict individual cognitive trajectories with reasonable accuracy, leading to uncertainty about future cognitive performance.

The currently known neural correlates of EF mainly comprise connectivity and network-based variables, reflecting the distributed brain networks involved. Functional brain connectivity refers to the statistical interdependencies between brain activity of network nodes (e.g. brain regions (Aertsen et al., 1989; Friston, 1994)). Network theory can assess local and global properties of the brain network (Bassett & Sporns, 2017; Sporns et al., 2005; Stam & Reijneveld, 2007; van den Heuvel & Hulshoff Pol, 2010). Cognition in general, and EF particularly, depends on long-distance integration across distributed brain regions, which can be operationalized through the centrality of regions within cognitively relevant networks (Baum et al., 2017; Deco et al., 2021; Medaglia et al., 2015; Sauseng et al., 2005).

In glioma, functional MRI-based connectivity of the frontoparietal network (FPN) is relevant for EF (Cochereau et al., 2020; Kocher et al., 2020; Landers et al., 2021; Lang et al., 2017; Maesawa et al., 2015; Noll et al., 2015, 2021; Tordjman et al., 2021). Using magnetoencephalography (MEG), frequency-specific connectivity has also been linked to EF in these patients: poorer EF at diagnosis relates to lower alpha band (8-13 Hz) functional connectivity (Derks et al., 2019), and particularly lower integrative connectivity in the theta (4-8 Hz), alpha, and beta (13-30 Hz) bands associate with poorer EF cross-sectionally (Bosma et al., 2009) and longitudinally (Carbo et al., 2017; van Dellen et al., 2012a).

Previous studies constructed separate functional networks for each frequency band, which may relate to particular cognitive and EF aspects, e.g. alpha band oscillations to attention (Klimesch, 2012) and theta and gamma band (coupling) to working memory (Kavanaugh et al., 2019). In EF, multiple cognitive aspects are combined, theoretically rendering it a relevant domain for frequency-integrated investigations. The multilayer network approach can be used to synergize frequencies, by defining a network within each frequency (layer), and then coupling these layers (De Domenico et al., 2013). The same nodes (i.e. brain regions) are present in each layer (Brookes et al., 2016; De Domenico et al., 2016; Guillon et al., 2017; Tewarie et al., 2016). Indeed, lower multilayer centrality of the FPN (including MEG and MRI layers) related to poorer EF in healthy subjects (Breedt et al., 2021) and lower centrality of the default mode network/hippocampus to poorer Mini Mental State Exam scores in Alzheimer’s disease (Yu et al., 2017).

In this study, we tested the hypotheses that (1) lower multilayer FPN integration correlates with poorer EF at diagnosis, and (2) changes in EF and multilayer centrality coincide. Finally, we hypothesized (3) lower multilayer centrality at diagnosis to predict deteriorating EF after tumor resection.

## Methods

### Patients

Patients between 2011 and 2021 at Amsterdam UMC with suspected diffuse glioma were eligible for participation in an ongoing prospective study on brain networks. Exclusion criteria were (1) age < 18 years, (2) psychiatric disease, (3) central nervous system comorbidities, (4) insufficient mastery of the Dutch language, and (5) inability to communicate adequately. After resection, molecular characteristics were assessed as part of clinical routine, including prognostically favorable isocitrate dehydrogenase (IDH) mutations and 1p/19q codeletions (Louis et al., 2021). This led to three subgroups: IDH-wildtype glioblastoma, IDH-mutant, non-codeleted astrocytoma, and IDH-mutant, 1p/19q-codeleted oligodendroglioma.

Patients underwent neuropsychological assessments (NPA (Derks et al., 2019)) and MEG at two time points: preoperatively at diagnosis (T1), and approximately one year after tumor resection (T2). Follow-up MEG and NPA took place between 8 and 20 months after resection, with a maximum of 3 months between them.

The current analysis was preregistered before selecting eligible patients and performing analyses (https://osf.io/83tbq).

The VUmc Medical Ethical Committee approved this study, which was conducted according to the Declaration of Helsinki. All participants provided written informed consent before participation.

### Neuropsychological assessment

Three EF tests were performed: the Categoric Word Fluency test (Mulder et al., 2006) for lexical access and updating aspects of EF, the Concept Shifting Test (van der Elst et al., 2006) for attention, working memory and set shifting, and the Stroop Color-Word Test (Hammes, 1978) for attention and inhibition (Supplementary Materials). Each test yielded one final score, which was adjusted for age, sex, and educational level and converted to a Z-score using validated normative data. We used a common cut-off value of Z < -1.5 to indicate cognitive impairment (Lezak, 2004).

### Magnetoencephalography

MEG was recorded for 5 min in supine position during eyes closed no-task resting-state in a magnetically shielded room (VacuumSchmelze GmBh, Hanau, Germany), using a 306-channel (102 magnetometers, 204 gradiometers) whole-head MEG system (Elekta Neuromag Oy, Helsinki, Finland) and a sampling frequency of 1250 Hz. Anti-aliasing (410 Hz) and high-pass filters (0.1 Hz) were applied online.

Preprocessing involved visual inspection, noisy channel removal, and noise removal in the remaining signals (see Supplementary Materials). Anatomical MRI was used for co-registration with the digitized scalp surface, and the Automated Anatomical Labeling atlas (Tzourio-Mazoyer et al., 2002) for parcellation of the cortical ribbon into 78 regions.

Broadband time series of neuronal activity were then reconstructed for each region’s centroid (Hillebrand et al., 2016) using a scalar beamformer approach (Hillebrand et al., 2012). For each patient, we included the first 60 epochs of 4096 samples (3.28s; total > 3 min). Fast Fourier transforms filtered the time series into six frequency bands: delta (0.5-4 Hz), theta (4-8 Hz), lower alpha (8-10 Hz), upper alpha (10-13 Hz), beta (13-30 Hz), and gamma (30-48 Hz). Finally, we computed and averaged the phase lag index (PLI (Stam et al., 2007)) between the frequency-filtered time series of region pairs using custom-made MATLAB scripts (R2020b, MathWorks, Natick, MA, USA; see https://github.com/multinetlab-amsterdam for code and data), yielding a single weighted network per frequency band per patient per time point.

### Multilayer network analysis

We used the multiplex network (Bianconi, 2018), which contains interlayer links with unitary weight only between the same nodes or brain regions across layers. Since differences in weight distribution across network layers impact multilayer network topology (Mandke et al., 2018), we first binarized each layer separately through Kruskal’s algorithm to construct minimum spanning trees (MSTs (Stam et al., 2014; Tewarie et al., 2015)). The MST recapitulates the network’s backbone, by incorporating each node and optimizing the overall weight of the network with n-1 connections (here 77) and no cycles. The MST has been amply applied in MEG, with a recent meta-analysis revealing consistent transdiagnostic MST alterations (Blomsma et al., 2022).

**Fig. 1:**
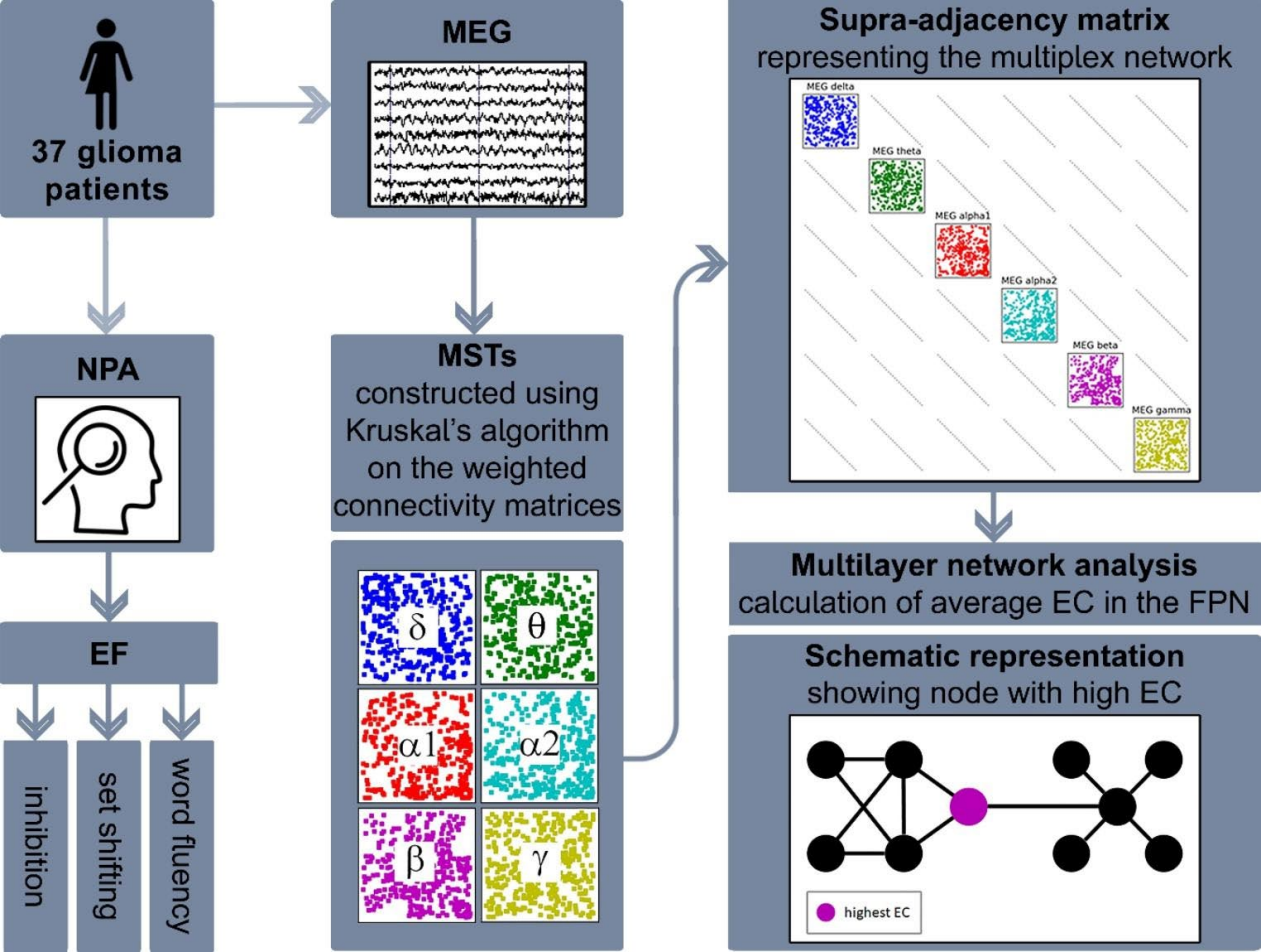
Schematic overview of the MEG multilayer analysis pipeline. For every participant, magnetoencephalography (MEG) data was preprocessed and projected to the brain; the brain was parcellated according to the Automated Anatomical Labeling (AAL) atlas; the Phase Lag Index (PLI) was used to compute weighted connectivity matrices; minimum spanning trees (MST) of the weighted matrices were constructed using Kruskal’s algorithm; and finally an LxN by LxN supra-adjacency matrix representing a multilayer network of the six MEG frequency bands as layers (all with N = 78 nodes for each AAL region and M = N – 1 = 77 binary intralayer links) was constructed, where diagonal blocks contain the intralayer connections for each frequency band and the off-diagonal blocks the interlayer connections; like the intralayer connections, we set all interlayer link weights to 1, obtaining binary multilayer networks; now, multilayer eigenvector centrality (EC) of the frontoparietal network (FPN) was calculated and averaged for each patient and timepoint. NPA = neuropsychological assessment, EF = executive functioning

We then calculated nodal multilayer eigenvector centrality (EC) as topological network measure of integration according to De Domenico et al. (2016) in Python (version 3.6, Python Software Foundation). EC takes the number of connections of a node and their neighboring nodes into account (Lohmann et al., 2010). EC relates to cognition in MEG literature (Hardmeier et al., 2012) and to EF in particular when using the multilayer approach (Breedt et al., 2021). Finally, nodal EC values of FPN nodes (according to (Yeo et al., 2011); Supplementary Table 1) were averaged, resulting in one multilayer FPN EC value per patient per time point (Fig. 1).

### Statistical analysis

Statistical analyses were performed using IBM SPSS (version 26.0, IBM Corp, Armonk, NY, USA). Paired t-tests (or nonparametric Wilcoxon signed-rank tests) assessed cognitive and network changes.

Backward linear regressions tested whether multilayer EC associated with EF at T1. For each EF aspect, a separate regression was performed. Based on literature (Derks et al., 2019; van Kessel et al., 2017; Wefel et al., 2016), glioma subtype and tumor location were selected as covariates. Potential additional covariates were selected through significant (*p* < 0.05) associations with the dependent variable (through correlation coefficients for age, time between resection and T2 NPA/MEG, tumor volume, percent tumor overlap with FPN; through t-tests or Mann-Whitney U tests for tumor lateralization, presence of epilepsy, education, sex, handedness, active treatment during or < 4 weeks before T2; through (Kruskal-Wallis) ANOVA for molecular subtype). Patients with tumor progression before T2 were only included for this cross-sectional T1 analysis.

To test whether multilayer EC changes (T2-T1) related to cognitive changes (T2-T1), backward linear regressions were performed. Based on literature (Hilverda et al., 2010; Koutsarnakis et al., 2021), type of anti-tumor treatment (radiotherapy, chemotherapy, chemoradiation, or no treatment) was included as covariate.

Backward linear regressions tested whether T1 multilayer EC predicted changes in EF. Again, anti-tumor treatment was included as covariate, and active treatment was explored as an additional covariate.

The level of significance was set at *p* < 0.05, and after Bonferroni correction at *p <* 0.0167 (p-value divided by three for each EF aspect).

## Results

### Patient characteristics

Thirty-seven participants (mean age 41.7 years ± SD 12.3) completed NPA and MEG at T1 and T2 (Table 1, see Supplementary Fig. 1 for lesion map). For two patients, the T1 set shifting score could not be calculated due to missing motor scores; three patients had incomplete T2 inhibition scores; one patient had a missing T2 word fluency score. In 4 patients, the interval between T2 MEG and NPA was > 3 months (range 4–7), but they were included as they had stable disease for at least 17 months after surgery.

**Table 1:** Patient characteristics

			Glioma patients (n = 37)	
Sex (males/females)			29/8	
Age in years (mean ± SD)			41.7 ± 12.3	
Education Verhage score (median (range))*			6 (4–7)	
Karnofsky Performance Status (KPS, median (range))			100 (80–100)	
Epilepsy (yes/no)**			33/4	
Use of antiepileptic drugs (yes/no)			32/5	
Interval resection-MEG in months (median (range))			12 (8–20)	
Interval resection-NPA in months (median (range))			12 (7–20)	
Handedness (left/right)			9/28	
Postoperative treatment before T2				
* Radiotherapy (n)*			4	
* Concomittant chemoradiation followed by adjuvant temozolomide (n)*			6	
* Radiotherapy followed by adjuvant chemotherapy (n)*			14	
* None (n)*			13	
Active chemotherapy during or < 4 weeks before T2 (n)			6	
Progression before T2 (yes/no)			7/30	
Tumor grade (II/III/IV)			23/8/6	
Tumor lateralization (left/right/bilateral)			25/11/1	
Location				
* Frontal (n)*			16	
* Temporal (n)*			9	
* Parietal (n)*			7	
* Frontotemporal (n)*			3	
* Insular (n)*			1	
* Occipital (n)*			1	
Molecular subtype				
* Glioblastoma, IDH-wildtype glioblastoma (n)*			6	
* Astrocytoma, IDH-mutant, non-codeleted (n)*			14	
* Oligodendroglioma, IDH-mutant, 1p/19q-codeleted (n)*			16	
* Astrocytoma, molecular subtype not available (n)*			1	
Tumor volume corrected for head size in mL (median (range))			34.26 (1.78–118.81)	
Tumor overlap with frontoparietal network in percent (median (range))			2.47 (0–28.1)	
T1 Multilayer centrality (mean ± SD)			0.41 ± 0.10	
T2 Multilayer centrality (mean ± SD)			0.42 ± 0.10	
Set shifting test completed (*n* at T1/T2)			35/37	
Word fluency test completed (*n* at T1/T2)			37/36	
Inhibition test completed (*n* at T1/T2)			37/34	

*Verhage et al., 1964. **Having epilepsy was assessed at T1, none of the participants developed seizures between T1 and T2.

**Fig. 2:**
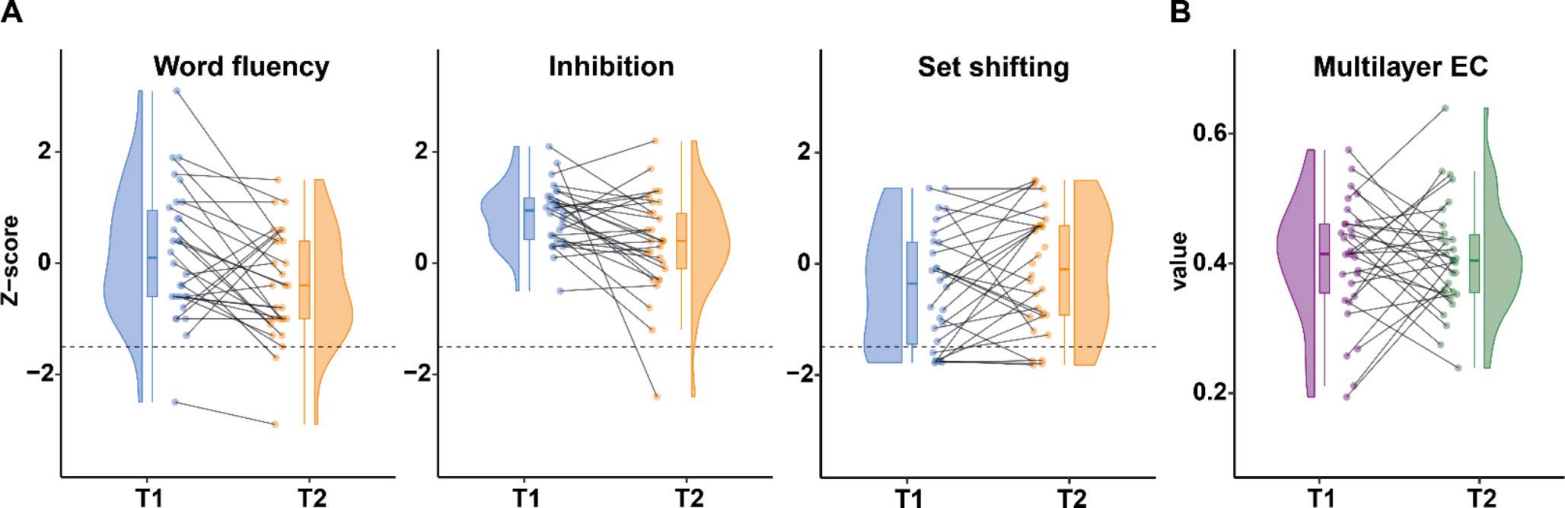
Executive functioning and multilayer integration at both time points. Each panel shows a paired raincloud plot, in which individual data points of each patient at both time points are displayed through the combination of a scatterplot (the ‘rain’), a spaghetti plot, a box plot, and a probability density plot (the ‘cloud’). Panel A shows patients’ Z-scores of the three executive functioning tests. Scores below the dashed line at -1.5 indicate clinically relevant cognitive deficits. Only word fluency changed significantly at the group-level (*p* = 0.002). Panel B shows patients’ multilayer eigenvector centrality (EC) of the frontoparietal network, which did not change significantly at the group-level.

At T1, 8 patients (22%) showed impaired set shifting (Fig. 2A); 2 patients (5%) had impaired word fluency; no patients had impaired inhibition. Longitudinally, 4 patients (11%) became unimpaired and 3 patients (8%) became impaired in set shifting; 4 patients (11%) declined to impaired word fluency; 1 patient (3%) became impaired in inhibition.

At the group-level, word fluency declined significantly and survived Bonferroni correction (*p* = 0.002, Fig. 2A). Inhibition declined non-significantly (*p* = 0.057). Set shifting remained unchanged (*p =* 0.441, Supplementary Table 2 shows individual scores, Supplementary Table 3 shows changes exceeding (-)1 Z-score).

At the group-level, multilayer EC did not change (*p =* 0.755, Table 1; Fig. 2B, Supplementary Fig. 2 visually represents molecular subtypes).

### Baseline brain-cognition correlations

**Fig. 3:**
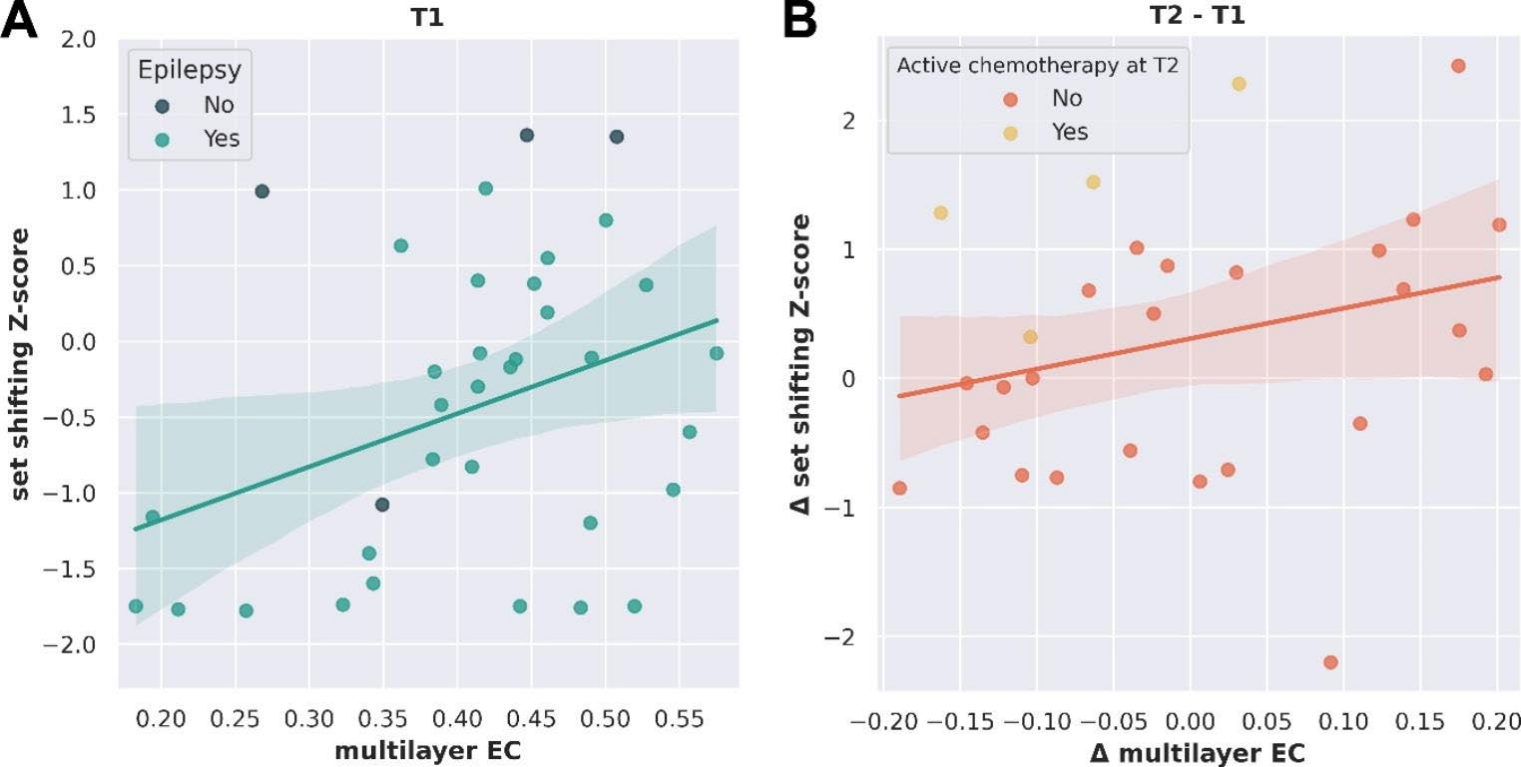
Significant associations between multilayer integration and set shifting. (A) Displays the cross-sectional association between multilayer eigenvector centrality (EC) of the frontoparietal network and set shifting performance (n = 35, model *p* = 0.003), with color codes indicating the included covariate (presence of epilepsy). (B) Shows the longitudinal association between change in multilayer EC (T2-T1) and change in set shifting (T2-T1; n = 28, model *p* = 0.006), with color codes indicating the included covariate (active chemotherapy at T2). Shaded area: 95% confidence interval.

For set shifting, having epilepsy or not was included as an additional covariate at T1, whereas no additional covariates were selected for word fluency and inhibition. Lower multilayer EC and having epilepsy significantly related to poorer set shifting after Bonferroni correction (*p* = 0.017 and *p* = 0.006 respectively, model *p* = 0.003; Table 2; Fig. 3A). Multilayer EC was not included in the final models for word fluency and inhibition, but patients with IDH-mutant, 1p/19q-codeleted glioma had better word fluency than IDH-wildtype glioblastoma patients (Supplementary Table 4).

**Table 2:** Significant regression results concerning multilayer integration

Dependent		adj. R^2^	F (df)	*p*-value	Significant predictors	β (stand.)	*p*-value	Excluded variables	*p*-value
T1 Set shifting		0.269	7.06 (2,31)	0.003*	T1 multilayer EC	0.377	0.017	Non-frontal tumor (ref = frontal)	0.970
					Presence of epilepsy (ref = no epilepsy)	-0.437	0.006	IDH-wildtype (ref = IDH-mutant, 1p/19q-codeleted)	0.470
								IDH-mutant, non-codeleted	0.324
Δ Set shifting		0.283	6.33 (2,25)	0.006*	Δ multilayer EC	0.414	0.022	Interval resection-NPA	0.524
					Active treatment at T2 (ref = no active treatment)	0.530	0.004		

*significant p-value (< 0.0167) after Bonferroni correction. EC = eigenvector centrality, Δ = change score (T2-T1), NPA = neuropsychological assessment.

### Longitudinal brain-cognition correlations

Longitudinal regression analyses were performed in patients without tumor progression before T2 (n = 30). At T2, six patients received active chemotherapy, whereas none received active radiotherapy or concomitant chemoradiation. For set shifting, active chemotherapy, resection-NPA interval, and resection-MEG interval correlated with change scores. Due to multicollinearity, only active chemotherapy (not anti-tumor treatment) and resection-NPA interval (not resection-MEG interval) were included. Decreasing multilayer EC and no active chemotherapy were associated with declining set shifting (*p =* 0.022 and *p =* 0.004, respectively, model *p =* 0.006; Table 2; Fig. 3B).

Multilayer EC changes did not relate to changes in inhibition and word fluency (Supplementary Table 4). Patients with IDH-mutant, 1p/19q-codeleted glioma showed greater word fluency decline than IDH-mutant, non-codeleted glioma patients (*p =* 0.001, Supplementary Fig. 3) and IDH-wildtype glioblastoma (*p =* 0.005, model *p* < 0.001).

### Baseline predictors of cognitive change

T1 multilayer EC did not predict cognitive change (Supplementary Table 4). Instead, better baseline inhibition and word fluency were associated with declining performance (*p =* 0.014 and *p* < 0.001, respectively, model *p* = 0.022 and *p* < 0.001, respectively). The model for inhibition did not survive correction for multiple testing. For set shifting, better baseline performance and no active chemotherapy were related to declining scores (model *p* = 0.016), but individual predictors did not reach significance (*p* = 0.069 and *p* = 0.091, respectively).

## Discussion

As expected, a proportion of glioma patients showed executive dysfunction, both at diagnosis and after tumor resection. While there were group-level decreases in word fluency only, we found variable individual trajectories for inhibition and set shifting. Partly confirming our hypotheses, lower and decreasing multilayer FPN integration related to poorer and deteriorating set shifting, but not word fluency and inhibition. Contrary to our hypothesis, baseline multilayer centrality did not predict postoperative changes in executive functioning.

Our observed association between multilayer integration and set shifting are in line with previous findings in healthy controls (Breedt et al., 2021), although that study used a composite EF score. Our results also corroborate work in other patient populations (Baggio et al., 2015; Liu et al., 2021; Marchesi et al., 2022), together suggesting that multilayer FPN integration is a general correlate of EF that is preserved in glioma. Central nodes like those within the FPN are thought to facilitate global communication between segregated communities, presumably enabling EF (Bertolero et al., 2017; Sporns, 2013). Our multilayer analysis indeed detects relevant individual EF variation in these patients, explaining ~ 27% of the cognitive variance in set shifting at diagnosis. Furthermore, our findings synthesize previous frequency-specific findings, where pathologically high local connectivity and clustering as well as lower integrative connectivity in specific frequency bands related to poorer cognitive performance (Bosma et al., 2009; Carbo et al., 2017; van Dellen et al., 2012a).

Correlations were significant only for set shifting, potentially reflecting particular sensitivity of multilayer integration towards this aspect of EF and not the other applied tests. Performance on the three tests showed very different distributions, and the large variability in set shifting performance over time may have led to greater statistical power to find correlations with multilayer integration. Certainly, the lack of findings for inhibition and word fluency could also be due to the small and heterogeneous sample. Hence, we should critically evaluate whether these findings are truly specific to set shifting in larger samples. Particularly, although we statistically adjusted for molecular subtype, scores on especially word fluency diverged between these subtypes. Moreover, higher T1 test scores predicted larger decline in word fluency, as also observed one year after epilepsy surgery (Vogt et al., 2018), potentially reflecting regression to the mean. As for covariates, having epilepsy was related to poorer set shifting at T1, congruent with earlier work (Klein et al., 2003). The finding that patients undergoing active chemotherapy at T2 showed improving set shifting remains difficult to interpret, particularly since none of these patients had shown tumor progression. Again, the relatively small sample should be kept in mind, particularly when it comes to the impact of epilepsy, treatment and tumor type on the dependent variables. The small number of patients representing each group may have led to spurious findings in terms of their impact on EF, and we refer readers to the wider literature when interpreting these results (see Supplementary Table 5 for an overview).

Contrary to our hypothesis, we did not find predictive significance of baseline multilayer integration towards EF change. Our current results suggest that, although relevant as a cross-sectional EF correlate in glioma (comparable to other populations), multilayer integration as operationalized here is not specifically relevant for glioma and may not predict EF trajectories in a heterogeneous glioma population.

There are several limitations that we considered when interpreting these results. Firstly, although large for a rare disease, our sample size is relatively small. Furthermore, we included a heterogeneous cohort, while molecular subtype particularly may pertain to both cognitive functioning (Wefel et al., 2016; Zhang et al., 2020) and network features (Jütten et al., 2020; Kesler et al., 2017). Some studies already indicated, like ours, that molecular subtypes may impact both cognition and network topology in a similar fashion, such that the actual brain-behavior associations are preserved across subtypes (Derks et al., 2019; Kesler et al., 2017). Future studies could include more patients of each subtype to enable more specific assessment of brain-cognition correlations within subgroups. This could potentially also reveal better predictors of cognitive decline. Thirdly, participation bias and therefore generalizability should always be considered in observational studies, as supported by our patients’ high performance status and generally maximal extent of resection, which is not representative of the entire glioma population. Fourthly, the multilayer network approach is new; other choices in terms of defining intra- and interlayer connectivity (Boccaletti et al., 2014) could be explored now that we established that multilayer integration relates to EF. Finally, we used a relatively coarse and non-connectivity-based atlas similar to earlier work (Carbo et al., 2017; Derks et al., 2019; van Dellen et al., 2012a; Dellen et al., 2012b), but replication with a connectivity-based atlas may yield additional and more specific results. Inevitably, tumor mass effects may still slightly affect native-to-template MRI registrations.

## Conclusion

We describe multilayer frequency-band integration of the frontoparietal network as a general correlate of executive functioning, also in glioma patients. Lower and decreasing integration related to poorer and declining set shifting. Baseline multilayer centrality did not have predictive value for declining EF over time. Still, our findings may inform future studies on the brain mechanisms underlying cognitive decline in these patients, as well as spark exploration of more targeted treatment of EF deficits.

## Electronic supplementary material

Below is the link to the electronic supplementary material.

Supplementary Material 1

## Data Availability

This analysis on existing data has been preregistered (https://osf.io/83tbq). Derivative data needed to replicate these results can be found at https://github.com/multinetlab-amsterdam.

## Code Availability

Scripts that were used to construct the supra-adjacency matrices in MATLAB and multilayer eigenvector centrality in the frontoparietal network in Python can be found here:

https://github.com/multinetlab-amsterdam.
