# Supplementary material for: The longitudinal relation between executive functioning and multilayer network topology in glioma patients
Source: Brain Imaging Behav. 2023 Apr 17;17(4):425–35. doi: 10.1007/s11682-023-00770-w (PMC10435610; doi:10.1007/s11682-023-00770-w)
Supplement: Supplementary file 1 — Supplementary Material 1 [file 11682_2023_770_MOESM1_ESM.pdf]

Supplementary Materials

The longitudinal relation between executive functioning and multilayer network topology in glioma patients

Marike R. van Lingen, Lucas C. Breedt, Jeroen J.G. Geurts, Arjan Hillebrand, Martin Klein, Mathilde C.M. Kouwenhoven, Shanna D. Kulik, Jaap C. Reijneveld, Cornelis J. Stam, Philip C. De Witt Hamer, Mona L.M. Zimmermann, Fernando A.N. Santos, Linda Douw

Methods

Patients

Part of this data has been reported on previously (Belgers et al., 2020; Carbo et al., 2017; Derks et al., 2018, 2019, 2021; Douw et al., 2008; van Dellen et al., 2012a, 2012b).

Because we applied the WHO tumor classification of 2021 (Louis et al., 2021) to a historical sample (diagnosed between 2010-2019), we re-interpreted the classification for some patients. We reassigned oligoastrocytomas that were IDH-mutant, 1p/19q-codeleted to oligodendrogliomas and IDH-mutant non-codeleted oligoastrocytomas to astrocytomas. If the IDH status or 1p/19q codeletion was not tested (n = 11), the molecular subtype was deduced from the clinical course (i.e. (progression free) survival) and histology. If available, we verified our classification based on their newest pathology rapport in case of a re-resection after T2.

Neuropsychological assessment

As this is the first study to relate EF to multilayer FPN integration in glioma, multiple domains of executive functioning were assessed by three different tests. The relation with multilayer FPN integration was analyzed for each test separately since we may find test-specific outcomes, as was the case for the shifting EF domain and FPN connectivity in a study in healthy subjects (Reineberg et al., 2018).

The goal of the Categoric Word Fluency Test (WFT (Mulder et al., 2006)) is for the participant to list, within 60 seconds, as many words as they can in a particular category. In the present study, we used the category ‘animals’. This semantic fluency test is widely used to assess updating aspects of executive functioning as well as working memory, inhibition and strategy (Henry & Crawford, 2004; Shao et al., 2014), although it also depends on a language component (Whiteside et al., 2016; Zigiotto et al., 2022).

The Concept Shifting Test (CST (Van der Elst et al., 2006)), an improved version of the Trail Making Test (TMT), comprises 16 small circles all of which contain a digit (part A), a letter (part B), or a letter or a digit (part C). The small circles are grouped into a larger circle. The goal of the test is for the participant to cross out the circles as fast as possible without making errors, in ascending (part A), alphabetical (part B), or alternating order (digit-letter; part C). Condition A and B require visual recognition and scanning, attention, long-term and working memory (to monitor the progress of the sequence). In condition C, the participant also needs to switch between the two sets (digits and letters) and must inhibit the response to continue within the same set (Van der Elst et al., 2006). To correct for motor speed, the participant additionally performs a dummy condition thrice, with empty circles.

The Stroop Color-Word Test (SCWT (Hammes, 1978)) consists of three cards that have to be read aloud by the participant. The first card contains the names of four colors (red, green, yellow, and blue) printed in black ink. The second card contains rectangles printed in these same colors. The third card contains the names of the four colors from the first card, but printed in an inconsistent color ink (e.g., the word 'blue' is printed in red ink) and the participant is told to ignore the word and list the color of the ink. All cards have to be read as fast as possible, without making mistakes.

Scoring

For the WFT, the total number of correct animals listed in 1 minute was used as the final score for the WFT. For the CST, the average time to complete the motor condition was subtracted from the time in condition A, B and C. The final outcome measure was the time in seconds on condition C, minus the average of condition A + B (after correcting for motor speed), to isolate the concept-shifting performance. Moreover, higher pre and postsurgical percentages of EF impairment have been reported for the TMT B-A score (the subtracted condition C score's equivalent) than for the TMT_B score in a study including 157 glioma patients (Lemaitre et al., 2021). For the SCWT, the interference score was calculated by subtracting the time in seconds of card 2 from the time on card 3, to isolate inhibition performance.

Raw scores for each subtest were then adjusted for sex, age, and education (classified according to the Dutch Verhage system (Verhage, 1964), which ranges from level 1 [less than six years of primary education] to level 7 [university degree]) by transforming them into Z-scores relative to population norms (Schmand et al., 2012; Van der Elst et al., 2006), yielding three EF Z-scores per patient. Since Z-score calculation may lead to uninterpretable outliers, especially when a patient is particularly slow, negative Z-scores were capped at Z = -3.0.

Magnetoencephalography

To aid visual inspection of the data, the cross-validation Signal Space Separation (SSS; (van Klink et al., 2017)) was applied, after which we (SDK, LD, LCB, MLMZ) removed at most 12 noisy channels. We used the temporal extension of SSS (Taulu & Simola, 2006) in MaxFilter (version 2.2.15) for further noise removal. Patients' head position was recorded continuously with head position coils, which were digitized together with the scalp shape using a 3D digitizer (Fastrak, Polhemus; Colchester, VT, USA). Anatomical MRI (GE Discovery 3T magnet, Milwaukee, USA; voxel size 1mm x 0.5mm x 0.5mm) was used for co-registration with the digitized scalp surface using a surface matching approach (estimated accuracy 4 mm (Whalen et al., 2008)). A single best-fitting sphere was fitted to the scalp outline and used as a volume-conductor model for the beamformer approach described below. We normalized the co-registered MRI to a template and, following inverse-normalization, labeled the voxels in the co-registered MRI according to the Automated Anatomical Labeling atlas (Tzourio-Mazoyer et al., 2002). Coregistration accuracy of the overlays of the atlas with native brains were visually inspected in each patient, excluding the presence of large artifacts related to altered anatomy and mass effects. Broadband time series of neuronal activity were then reconstructed for each region's centroid (Hillebrand et al., 2016) using a scalar beamformer approach (Hillebrand et al., 2012). The normalized (Cheyne et al., 2007) beamformer weights were based on the lead fields for dipolar sources, broadband (0.5-48Hz) data covariance, and unity noise covariance (for the estimation of the optimum orientation (Sekihara et al., 2004)). Anatomical MRI (T1-

weighted images with and without contrast injection and T2 or FLAIR) was used to segment the tumor for each individual patient, which was then coregistered to the Montreal Neurological Institute (MNI) template brain to create a lesion map (Supplementary Figure 1).

Supplementary Table 1. Regions of the frontoparietal network within the atlas

**Regions of the frontoparietal network**	X	Y	Z
Inferior frontal gyrus, orbital part, L	-35.98	30.71	-12.11
Inferior frontal gyrus, orbital part, R	41.22	32.23	-11.91
Superior frontal gyrus, L	-18.45	34.81	42.20
Superior frontal gyrus, R	21.90	31.12	43.82
Middle frontal gyrus, L	-33.43	32.73	35.46
Middle frontal gyrus, R	37.59	33.06	34.04
Superior parietal gyrus, L	-23.45	-59.56	58.96
Superior parietal gyrus, R	26.11	-59.18	62.06
Inferior parietal gyrus, L	-42.80	-45.82	46.74
Inferior parietal gyrus, R	46.46	-46.29	49.54
Middle cingulate gyrus, L	-5.48	-14.92	41.57
Middle cingulate gyrus, R	8.02	-8.83	39.79

Legend. L = left hemisphere, R = right hemisphere

Supplementary Table 2. Case summary of individual executive functioning corrected Z-scores at T1, T2 and over time (Δ).

Patients with (n = 7) and without (n = 30) progression before T2 are shown.

	T1 CST	T2 CST	Δ CST	T1 WFT	T2 WFT	Δ WFT	T1 SCWT	T2 SCWT	Δ SCWT
Progression									
1	-1.20	-1.20	0.00	-0.80	-1.30	-0.50	0.40	-0.80	-1.20
2	0.63	-1.74	-2.37	-1.00	-1.30	-0.30	0.90	-0.80	-1.70
3	0.37	0.30	-0.07	1.30	0.80	-0.50	-0.30	0.00	0.30
4	-0.11	-0.09	0.02	0.80	1.00	0.20	-0.50	1.10	1.60
5	-0.60	-0.24	0.36	-0.40	-1.90	-1.50	-0.40	2.00	2.40
6	-0.17	-1.80	-1.63	-0.80	-2.70	-1.90	-0.80	-	-
7	-1.75	-1.81	-0.06	-1.70	-2.30	-0.60	-0.80	-	-
N	7	7	7	7	7	7	7	5	5
Mean	-0.40	-0.94	-0.54	-0.37	-1.10	-0.73	-0.21	0.30	0.28
Min.	-1.75	-1.81	-2.37	-1.70	-2.70	-1.90	-0.80	-0.80	-1.70
Max.	0.63	0.30	0.36	1.30	1.00	0.20	0.90	2.00	2.40
SD	0.84	0.91	1.03	1.06	1.46	0.72	0.64	1.23	1.76
No progression									
1	-0.30	0.68	0.99	1.50	-1.00	-2.50	1.80	-2.40	-4.20
2	-1.78	0.64	2.42	-2.50	-2.90	-0.40	0.30	-1.20	-1.50
3	-	0.01	-	-0.60	-0.40	0.20	0.40	-0.80	-1.20
4	-0.83	0.69	1.52	-0.80	0.60	1.40	-0.50	-0.40	0.10
5	-1.76	-1.80	-0.04	-0.60	-0.80	-0.20	0.50	-0.30	-0.80
6	1.01	0.66	-0.35	0.40	-1.30	-1.70	1.10	-0.30	-1.40
7	-0.78	1.50	2.28	0.20	-1.70	-1.90	1.20	-0.30	-1.50
8	-1.60	-1.28	0.32	0.40	-0.40	-0.80	0.90	-0.10	-1.00
9	1.36	0.80	-0.56	1.00	0.00	-1.00	1.00	0.00	-1.00
10	1.35	1.35	0.00	-0.60	-1.00	-0.40	0.30	0.10	-0.20
11	-1.75	-1.82	-0.07	-0.40	0.60	1.00	0.40	0.20	-0.20
12	-0.20	-0.96	-0.75	-1.00	-1.00	0.00	0.70	0.20	-0.50
13	-0.08	-0.88	-0.80	3.10	0.40	-2.70	0.10	0.30	0.20
14	-1.74	-0.92	0.82	-1.00	-1.00	0.00	0.30	0.40	0.10

15	0.38	1.06	0.68	1.90	1.10	-0.80	1.10	0.40	-0.70
16	0.55	1.43	0.87	0.00	-1.00	-1.00	1.20	0.40	-0.80
17	-	0.30	-	0.40	-0.40	-0.80	1.30	0.40	-0.90
18	0.80	1.49	0.69	1.10	1.10	0.00	0.30	0.60	0.30
19	0.40	-0.31	-0.71	0.60	-1.10	-1.70	0.50	0.60	0.10
20	0.19	0.69	0.50	1.90	-0.20	-2.10	1.10	0.80	-0.30
21	-0.98	-1.75	-0.77	-0.60	-1.30	-0.70	1.30	0.90	-0.40
22	-0.12	-0.54	-0.42	-0.60	-0.80	-0.20	2.10	0.90	-1.20
23	-0.42	0.59	1.01	-1.30	0.40	1.70	0.90	1.10	0.20
24	-0.08	-0.93	-0.85	-1.00	0.60	1.60	1.40	1.10	-0.30
25	-1.40	-0.21	1.19	0.80	-	-	1.00	1.20	0.20
26	-1.08	0.15	1.23	1.10	0.40	-0.70	0.60	1.30	0.70
27	-1.75	-0.46	1.28	0.80	-1.00	-1.80	1.00	1.30	0.30
28	0.99	-1.21	-2.20	-0.20	0.00	0.20	0.80	1.70	0.90
29	-1.16	-0.80	0.37	1.60	1.50	-0.10	1.60	2.20	0.60
30	-1.77	-1.74	0.03	-0.40	-1.50	-1.10	1.00	-	-
N	28	30	28	30	29	29	30	29	29
Mean	-0.45	-0.12	0.31	0.17	-0.42	-0.57	0.86	0.36	-0.50
Min.	-1.78	-1.82	-2.20	-2.50	-2.90	-2.70	-0.50	-2.40	-4.20
Max.	1.36	1.50	2.42	3.10	1.50	1.70	2.10	2.20	0.90
SD	1.05	1.06	1.03	1.18	0.98	1.14	0.54	0.91	0.98

CST = Concept Shifting Test, WFT = Word Fluency Test, SCWT = Stroop Color-Word Test, SD = standard deviation.

Supplementary Table 3. Overview of longitudinal cognitive Z-score changes of (-)1 or more.

	All patients		Patients without progression	
	1 ≥ Δ Z-score ↑	-1 ≤ Δ Z-score ↓	1 ≥ Δ Z-score ↑	-1 ≤ Δ Z-score ↓
Fluency n (%)	4 (11.1%)	12 (33.3%)	4 (13.8%)	10 (34.5%)
Set shifting n (%)	7 (20.0%)	3 (8.6%)	7 (25.0%)	1 (3.6%)
Inhibition n (%)	2 (5.9%)	10 (29.4%)	0 (0%)	8 (27.6%)

Supplementary Table 4. Non-significant results concerning multilayer integration

Dependent	adj. R^2	F (df)	*p*-value	Included predictors	B (stand.)	*p*-value	Excluded variables	*p*-value
T1 Word fluency	0.135	3.73 (2,33)	0.035	IDH-wildtype (ref = IDH-mutant, 1p/19q-codeleted)	-0.430	0.015	T1 multilayer EC	0.451
				IDH-mutant, non-codeleted	-0.305	0.079	Non-frontal tumor (ref = frontal)	0.126
T1 Inhibition	0.060	3.25 (1,34)	0.080	IDH-wildtype	-0.295	0.080	Non-frontal tumor	0.709
							IDH-mutant, non-codeleted	0.575
							T1 multilayer EC	0.533
Δ Word fluency	0.396	9.84 (2,25)	<0.001*	IDH-wildtype	0.482	0.005	Δ multilayer EC	0.827
				IDH-mutant, non-codeleted	0.626	0.001	RT and XT	0.519
Δ Inhibition	-0.013	0.652 (1,27)	0.426	RT (ref = no treatment)	-0.154	0.426	Δ multilayer EC	0.885
							RT and XT	0.645
Δ Word fluency	0.617	15.5 (3,24)	<0.001*	T1 Word fluency	-0.498	0.001	T1 multilayer EC	0.743
				IDH-wildtype	0.327	0.020	RT	0.946
				IDH-mutant, non-codeleted	0.491	0.001	RT and XT	0.195
Δ Inhibition	0.197	4.44 (2,26)	0.022	T1 Inhibition	-0.452	0.014	T1 multilayer EC	0.812

				Age	-0.309	0.084	RT and XT	0.599
							RT	0.167
Δ Set shifting	0.223	4.88 (2,25)	0.016*	T1 Set shifting	-0.340	0.069	T1 multilayer EC	0.766
				Active treatment at T2 (ref = no treatment)	0.341	0.091	Interval resection - NPA	0.660

*significant p-value (<.0167) after Bonferroni correction. EC = eigenvector centrality, Δ = change score (T2-T1), RT = radiotherapy, XT = chemotherapy, NPA = neuropsychological assessment. In case of dummy-coded variables, the reference category (ref) is indicated at first mention only.

Supplementary Table 5. Selection of relevant literature on cognition in glioma before and after resective surgery

	References	Type
**Presurgical cognitive functioning**	(Caramanna et al., 2021)	Research article
	(van Kessel et al., 2017)	Systematic review
	(Wefel et al., 2016)	Research article
	(Zhang et al., 2020)	Research article
**Pre- and postsurgical cognitive functioning**	(Cochereau et al., 2020)	Research article
	(Klein et al., 2004)	Research article
	(Klein et al., 2012)	Review
	(Lemaitre et al., 2021)	Research article
	(Ng et al., 2019)	Meta-analysis
	(Sinha et al., 2020)	Systematic review
	(Tabor et al., 2021)	Review
	(Tanzilli et al., 2022)	Research article

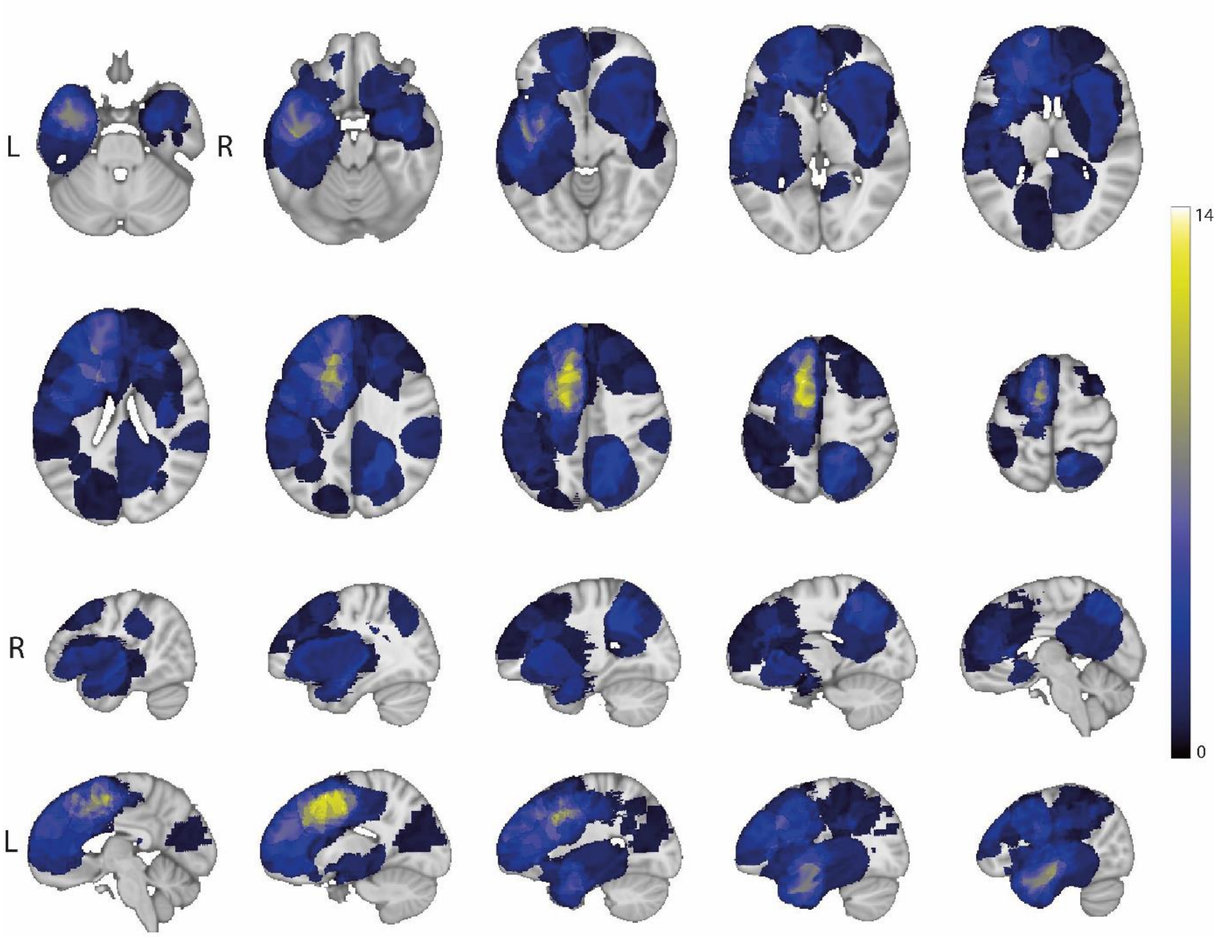

Supplementary Figure 1. Lesion map

Low tumor occurrence is indicated with blue colors and high occurrence with yellow colors (maximum is n = 14 patients). A preference for the left hemisphere is observed.

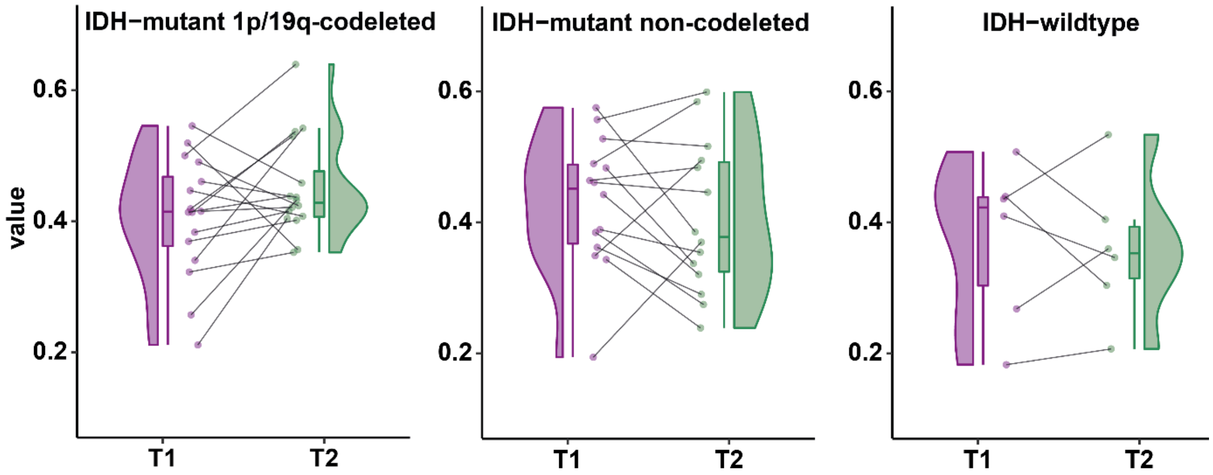

Supplementary Figure 2. Individual changes in multilayer integration per glioma subtype

Each panel shows multilayer eigenvector centrality of the frontoparietal network in patients with IDH-mutant 1p/19q-codeleted, IDH-mutant non-codeleted, and IDH-wildtype gliomas at both time points.

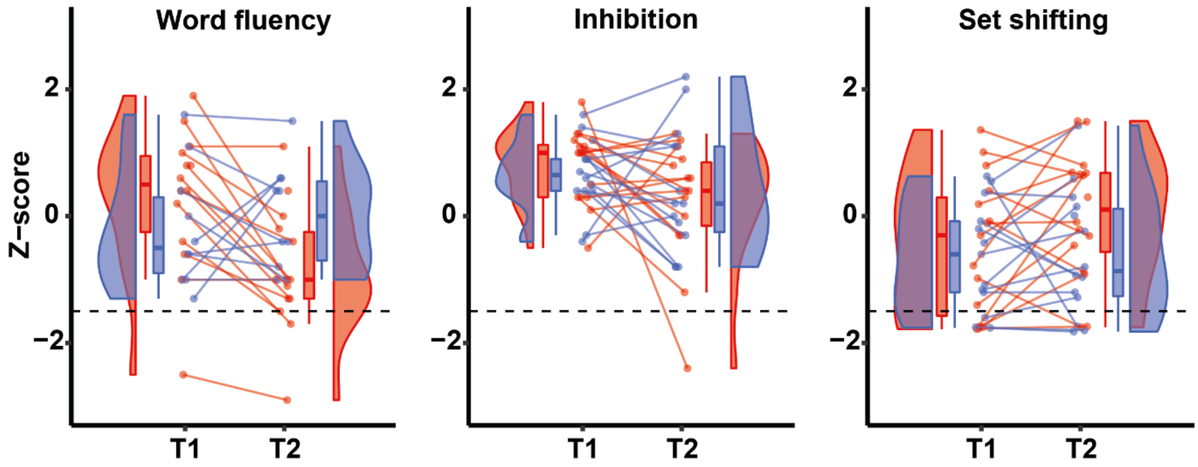

Supplementary Figure 3. Individual changes in executive functioning at both time points

The raincloud plots with clouds, raindrops and lines represent the IDH-mutant non-codeleted (blue) and IDH-mutant 1p/19q-codeleted (red) glioma subgroup. Scores below the dashed line at -1.5 indicate clinically relevant cognitive deficits.

References

Belgers, V., Numan, T., Kulik, S. D., Hillebrand, A., de Witt Hamer, P. C., Geurts, J. J. G., Reijneveld, J. C., Wesseling, P., Klein, M., Derks, J., & Douw, L. (2020). Postoperative oscillatory brain activity as an add-on prognostic marker in diffuse glioma. *J Neurooncol*, *147*(1), 49-58. https://doi.org/10.1007/s11060-019-03386-7

Caramanna, I., Bottomley, A., Drijver, A. J., Twisk, J., van den Bent, M., Idbaih, A., Wick, W., Pe, M., Klein, M., Reijneveld, J. C., Group, E. Q. o. L., & Brain Tumour, G. (2021). Objective neurocognitive functioning and neurocognitive complaints in patients with high-grade glioma: Evidence of cognitive awareness from the European Organisation for Research and Treatment of Cancer brain tumour clinical trials. *Eur J Cancer*, *144*, 162-168. https://doi.org/10.1016/j.ejca.2020.10.040

Carbo, E. W., Hillebrand, A., van Dellen, E., Tewarie, P., de Witt Hamer, P. C., Baayen, J. C., Klein, M., Geurts, J. J., Reijneveld, J. C., Stam, C. J., & Douw, L. (2017). Dynamic hub load predicts cognitive decline after resective neurosurgery. *Sci Rep*, *7*, 42117. https://doi.org/10.1038/srep42117

Cheyne, D., Bostan, A. C., Gaetz, W., & Pang, E. W. (2007). Event-related beamforming: a robust method for presurgical functional mapping using MEG. *Clinical Neurophysiology*, *118*(8), 1691-1704.

Cochereau, J., Lemaitre, A. L., Wager, M., Moritz-Gasser, S., Duffau, H., & Herbet, G. (2020). Network-behavior mapping of lasting executive impairments after low-grade glioma surgery. *Brain Struct Funct*, *225*(8), 2415-2429. https://doi.org/10.1007/s00429-020-02131-5

Derks, J., Kulik, S., Wesseling, P., Numan, T., Hillebrand, A., van Dellen, E., de Witt Hamer, P. C., Geurts, J. J. G., Reijneveld, J. C., Stam, C. J., Klein, M., & Douw, L. (2019). Understanding cognitive functioning in glioma patients: The relevance of IDH-mutation status and functional connectivity. *Brain Behav*, *9*(4), e01204. https://doi.org/10.1002/brb3.1204

Derks, J., Kulik, S. D., Numan, T., de Witt Hamer, P. C., Noske, D. P., Klein, M., Geurts, J. J. G., Reijneveld, J. C., Stam, C. J., Schoonheim, M. M., Hillebrand, A., & Douw, L. (2021). Understanding Global Brain Network Alterations in Glioma Patients. *Brain Connect*, *11*(10), 865-874. https://doi.org/10.1089/brain.2020.0801

Derks, J., Wesseling, P., Carbo, E. W. S., Hillebrand, A., van Dellen, E., de Witt Hamer, P. C., Klein, M., Schenk, G. J., Geurts, J. J. G., Reijneveld, J. C., & Douw, L. (2018). Oscillatory brain activity associates with neuroligin-3 expression and predicts progression free survival in patients with diffuse glioma. *J Neurooncol*, *140*(2), 403-412. https://doi.org/10.1007/s11060-018-2967-5

Douw, L., Baayen, H., Bosma, I., Klein, M., Vandertop, P., Heimans, J., Stam, K., de Munck, J., & Reijneveld, J. (2008). Treatment-related changes in functional connectivity in brain tumor patients: a magnetoencephalography study. *Exp Neurol*, *212*(2), 285-290. https://doi.org/10.1016/j.expneurol.2008.03.013

Hammes, J. G. W. (1978). *De Stroop kleur-woord test*. Harcourt Test Publ.

Henry, J. D., & Crawford, J. R. (2004). Verbal fluency deficits in Parkinson's disease: a meta-analysis. *J Int Neuropsychol Soc*, *10*(4), 608-622. https://doi.org/10.1017/S1355617704104141

Hillebrand, A., Barnes, G. R., Bosboom, J. L., Berendse, H. W., & Stam, C. J. (2012). Frequency-dependent functional connectivity within resting-state networks: an atlas-based MEG beamformer solution. *Neuroimage*, *59*(4), 3909-3921. https://doi.org/10.1016/j.neuroimage.2011.11.005

Hillebrand, A., Tewarie, P., van Dellen, E., Yu, M. C., Carbo, E. W. S., Douw, L., Gouw, A. A., van Straaten, E. C. W., & Stam, C. J. (2016). Direction of information flow in large-scale resting-state networks is frequency-dependent. *Proceedings of the National Academy of Sciences of the United States of America*, *113*(14), 3867-3872. https://doi.org/10.1073/pnas.1515657113

Klein, M., Duffau, H., & De Witt Hamer, P. C. (2012). Cognition and resective surgery for diffuse infiltrative glioma: an overview. *J Neurooncol*, *108*(2), 309-318. https://doi.org/10.1007/s11060-012-0811-x

Klein, M., Heimans, J., Aaronson, N., Postma, T., Muller, M., Van der Ploeg, H., & Taphoorn, M. (2004). Impaired cognitive functioning in low-grade glioma patients: relationship to tumor localisation, radiotherapy and the use of anticonvulsants. *Nederlands Tijdschrift voor Geneeskunde*, *148*(44), 2175-2180.

Lemaitre, A. L., Herbet, G., Ng, S., Moritz-Gasser, S., & Duffau, H. (2021). Cognitive preservation following awake mapping-based neurosurgery for low-grade gliomas: a longitudinal, within-patient design study. *Neuro Oncol*. https://doi.org/10.1093/neuonc/noab275

Louis, D. N., Perry, A., Wesseling, P., Brat, D. J., Cree, I. A., Figarella-Branger, D., Hawkins, C., Ng, H. K., Pfister, S. M., Reifenberger, G., Soffietti, R., von Deimling, A., & Ellison, D. W. (2021). The 2021 WHO Classification of Tumors of the Central Nervous System: a summary. *Neuro Oncol*, *23*(8), 1231-1251. https://doi.org/10.1093/neuonc/noab106

Mulder, J., Dekker, P., & Dekker, R. (2006). *Woord-fluency test/figuur-fluency test, handleiding*. PITS.

Ng, J. C. H., See, A. A. Q., Ang, T. Y., Tan, L. Y. R., Ang, B. T., & King, N. K. K. (2019). Effects of surgery on neurocognitive function in patients with glioma: a meta-analysis of immediate post-operative and long-term follow-up neurocognitive outcomes. *J Neurooncol*, *141*(1), 167-182. https://doi.org/10.1007/s11060-018-03023-9

Reineberg, A. E., Gustavson, D. E., Benca, C., Banich, M. T., & Friedman, N. P. (2018). The Relationship Between Resting State Network Connectivity and Individual Differences in Executive Functions. *Front Psychol*, *9*, 1600. https://doi.org/10.3389/fpsyg.2018.01600

Schmand, B., Houx, P., & De Koning, I. (2012). Normen van psychologische tests voor gebruik in de klinische neuropsychologie. *Sectie Neuropsychologie Nederlands Instituut van Psychologen*.

Sekihara, K., Nagarajan, S. S., Poeppel, D., & Marantz, A. (2004). Asymptotic SNR of scalar and vector minimum-variance beamformers for neuromagnetic source reconstruction. *IEEE transactions on biomedical engineering*, *51*(10), 1726-1734.

Shao, Z., Janse, E., Visser, K., & Meyer, A. S. (2014). What do verbal fluency tasks measure? Predictors of verbal fluency performance in older adults. *Front Psychol*, *5*, 772. https://doi.org/10.3389/fpsyg.2014.00772

Sinha, R., Stephenson, J. M., & Price, S. J. (2020). A systematic review of cognitive function in patients with glioblastoma undergoing surgery. *Neurooncol Pract*, *7*(2), 131-142. https://doi.org/10.1093/nop/npz018

Tabor, J. K., Bonda, D., LeMonda, B. C., & D'Amico, R. S. (2021). Neuropsychological outcomes following supratotal resection for high-grade glioma: a review. *J Neurooncol*, *152*(3), 429-437. https://doi.org/10.1007/s11060-021-03731-9

Tanzilli, A., Pace, A., Prosperini, L., Oppido, P. A., Telera, S., Rasile, F., Farneti, A., Marucci, L., & Villani, V. (2022). Multidomain and long-term cognitive evaluation in malignant glioma patients. *Future Oncol*, *18*(15), 1839-1848. https://doi.org/10.2217/fon-2021-0963

Taulu, S., & Simola, J. (2006). Spatiotemporal signal space separation method for rejecting nearby interference in MEG measurements. *Phys Med Biol*, *51*(7), 1759-1768. https://doi.org/10.1088/0031-9155/51/7/008

Tzourio-Mazoyer, N., Landeau, B., Papathanassiou, D., Crivello, F., Etard, O., Delcroix, N., Mazoyer, B., & Joliot, M. (2002). Automated anatomical labeling of activations in SPM using a macroscopic anatomical parcellation of the MNI MRI single-subject brain. *Neuroimage*, *15*(1), 273-289. https://doi.org/10.1006/nimg.2001.0978

van Dellen, E., de Witt Hamer, P. C., Douw, L., Klein, M., Heimans, J. J., Stam, C. J., Reijneveld, J. C., & Hillebrand, A. (2012a). Connectivity in MEG resting-state networks increases after resective surgery for low-grade glioma and correlates with improved cognitive performance. *Neuroimage Clin*, *2*, 1-7. https://doi.org/10.1016/j.nicl.2012.10.007

van Dellen, E., Douw, L., Hillebrand, A., Ris-Hilgersom, I. H., Schoonheim, M. M., Baayen, J. C., De Witt Hamer, P. C., Velis, D. N., Klein, M., Heimans, J. J., Stam, C. J., & Reijneveld, J. C. (2012b). MEG network differences between low- and high-grade glioma related to epilepsy and cognition. *PLoS One*, *7*(11), e50122. https://doi.org/10.1371/journal.pone.0050122

Van der Elst, W., Van Boxtel, M. P., Van Breukelen, G. J., & Jolles, J. (2006). The concept shifting test: Adult normative data. *Psychological assessment*, *18*(4), 424.

van Kessel, E., Baumfalk, A. E., van Zandvoort, M. J. E., Robe, P. A., & Snijders, T. J. (2017). Tumor-related neurocognitive dysfunction in patients with diffuse glioma: a systematic review of neurocognitive functioning prior to anti-tumor treatment. *J Neurooncol*, *134*(1), 9-18. https://doi.org/10.1007/s11060-017-2503-z

van Klink, N., van Rosmalen, F., Nenonen, J., Burnos, S., Helle, L., Taulu, S., Furlong, P. L., Zijlmans, M., & Hillebrand, A. (2017). Automatic detection and visualisation of MEG ripple oscillations in epilepsy. *Neuroimage Clin*, *15*, 689-701. https://doi.org/10.1016/j.nicl.2017.06.024

Wefel, J. S., Noll, K. R., Rao, G., & Cahill, D. P. (2016). Neurocognitive function varies by IDH1 genetic mutation status in patients with malignant glioma prior to surgical resection. *Neuro Oncol*, *18*(12), 1656-1663. https://doi.org/10.1093/neuonc/now165

Whalen, C., Maclin, E. L., Fabiani, M., & Gratton, G. (2008). Validation of a method for coregistering scalp recording locations with 3D structural MR images. *Hum Brain Mapp*, *29*(11), 1288-1301. https://doi.org/10.1002/hbm.20465

Whiteside, D. M., Kealey, T., Semla, M., Luu, H., Rice, L., Basso, M. R., & Roper, B. (2016). Verbal Fluency: Language or Executive Function Measure? *Appl Neuropsychol Adult*, *23*(1), 29-34. https://doi.org/10.1080/23279095.2015.1004574

Zhang, Z., Jin, Z., Yang, X., Zhang, L., Zhang, Y., Liu, D., Chi, X., Hao, S., Feng, J., & Ji, N. (2020). Pre-operative Neurocognitive Function Was More Susceptible to Decline in Isocitrate Dehydrogenase Wild-Type Subgroups of Lower-Grade Glioma Patients. *Front Neurol*, *11*, 591615. https://doi.org/10.3389/fneur.2020.591615

Zigiotto, L., Vavassori, L., Annicchiarico, L., Corsini, F., Avesani, P., Rozzanigo, U., Sarubbo, S., & Papagno, C. (2022). Segregated circuits for phonemic and semantic fluency: A novel patient-tailored disconnection study. *Neuroimage Clin*, *36*, 103149. https://doi.org/10.1016/j.nicl.2022.103149
